# Application of laboratory-result-oriented modular teaching model in the training of clinical hematology laboratory interns

**DOI:** 10.3389/fmed.2025.1612934

**Published:** 2025-08-18

**Authors:** Hui Xie, Jiale Li, Yijia Zhu, Lin Chen, Aofan Wang, Linyan Gong, Tingting Tan, Xian Wang, Yuxin Chen

**Affiliations:** ^1^Department of Laboratory Medicine, Nanjing Drum Tower Hospital Clinical College of Nanjing Medical University, Nanjing, China; ^2^Department of Laboratory Medicine, Nanjing Drum Tower Hospital Clinical College of Jiangsu University, Zhenjiang, China; ^3^Department of Laboratory Medicine, Nanjing Drum Tower Hospital Clinical College of Nanjing University of Chinese Medicine, Nanjing, China

**Keywords:** medical laboratory education, laboratory results, modular teaching, interns, teaching effectiveness

## Abstract

**Background:**

Current teaching models for clinical hematology laboratory interns often prioritize theoretical knowledge dissemination over the practical application of skills directly tied to analyzing and interpreting laboratory results. This approach potentially limits the development of critical clinical reasoning and problem-solving abilities within the framework of quality management systems like ISO 15189. To address these challenges, this study implemented and evaluated a novel laboratory-result-oriented modular teaching model, explicitly integrating ISO 15189 principles. The study assessed the design, implementation, effectiveness, advantages, and challenges of this model compared to traditional methods.

**Methods:**

A total of 60 medical laboratory interns were recruited. Thirty interns completed their internship from October 2022 to March 2023 (control group), following the traditional model involving theoretical lectures supplemented by limited laboratory demonstrations. The remaining 30 interns undertook their internship from October 2023 to March 2024 (experimental group), receiving the laboratory-result-oriented modular teaching. Teaching effectiveness was compared through theoretical examinations, operational skill assessments, and satisfaction surveys.

**Results:**

The experimental group scored significantly higher than the control group in both theoretical examinations and operational skill assessments (*p* < 0.05). Their overall teaching satisfaction was also significantly higher (*p* < 0.05).

**Conclusion:**

The laboratory-result-oriented modular teaching model improved teaching effectiveness and fostered greater learning initiative among interns. It enhanced interns’ ability to analyze and solve problems independently and developed clinical reasoning skills.

## Introduction

Clinical laboratory medicine is a highly practical and interdisciplinary field (1–3), requiring rigorous quality management systems (QMS) to ensure reliable diagnostic results (4). ISO 15189:2012 (*Medical Laboratories—Requirements for Quality and Competence*, hereafter ISO 15189) represents the international standard for accrediting medical laboratory QMS (5–7). This standard emphasizes personnel competence as paramount for laboratory quality. Consequently, training programs for interns should evolve beyond traditional models focused solely on technical skills by explicitly integrating ISO 15189 core principles. This integration aims to cultivate professionals possessing not only technical proficiency and research potential but also robust quality management awareness.

Clinical hematology testing is pivotal for diagnosis and patient management (8–13). The accuracy and interpretation of blood test results directly impact clinical decisions (14, 15). However, conventional teaching models, often instructor-centered with passive knowledge delivery and summative assessment (16), frequently fail to adequately develop the analytical, interpretative, and quality management skills required within accredited laboratories, particularly under the ISO 15189 framework.

To address this gap, we propose, implement, and evaluate a novel “Laboratory-Result-Oriented Modular Teaching Model based on ISO 15189 principles” for clinical hematology interns. This model utilizes actual laboratory test results (e.g., complete blood counts [CBCs], coagulation profiles) as the anchor for learning, stimulating inquiry into their generation, reliability, and clinical meaning. The curriculum is structured into discrete, focused modules, each dedicated to a specific result-related aspect such as pre-analytical variables (17–19), analytical methods (including instrument operation and quality control aligned with ISO 15189), quality control, result interpretation, or clinical case integration. Crucially, the model systematically embeds core ISO 15189 requirements—including personnel roles, procedural documentation, quality control processes, equipment management, and the principles of continuous improvement and patient safety—throughout these modules.

By integrating internship management within the ISO 15189 QMS framework using this result-oriented modular approach, students directly engage with standardized practices. This framework aims to link theory and practice directly to result reliability, preparing interns for regulated practice and enhancing future result accuracy (20, 21). The pedagogical strategy leverages test results to drive inquiry within defined, ISO 15189-compliant modules, aiming to improve clinical reasoning, problem-solving, and intrinsic quality awareness. To empirically evaluate this model’s effectiveness and feasibility compared to traditional teaching, a controlled study was conducted with two intern cohorts.

## Study subjects and methodology

### Study participants

A total of 60 medical laboratory interns from a tertiary Grade-A hospital in Nanjing were recruited. Thirty interns completing their internship from October 2022 to March 2023 were assigned to the control group, and 30 interns undertaking their internship from October 2023 to March 2024 comprised the experimental group. Inclusion criteria required enrollment as interns within the medical laboratory department. Exclusion criteria were: (a) internship interruption, and (b) absence from one or more teaching sessions. To mitigate potential confounding effects from non-synchronized internship periods (2022–2023 vs. 2023–2024), we confirmed: (a) an identical instructional team composition and laboratory equipment; (b) unchanged ISO 15189 operational protocols; (c) no institutional reforms affecting training structure; and (d) no significant inter-group differences in pre-internship scores (Table 1, *p* > 0.05). (e) All instructors were certified Medical Laboratory Quality and Capability Accreditation Criteria Project Training (Certification No.: NJGLJYK-21-06-037). All participants provided written informed consent after being fully informed of the study’s objectives and methods. Ethical approval was obtained from the Ethics Committee of Nanjing Drum Tower Hospital (Approval No: 2022–238-02).

**Table 1:** Demographic information.

Item	Control group (*n* = 30)	Experimental group (*n* = 30)	*p*
Gender			1.0
Male	15 (50%)	15 (50%)	
Female	15 (50%)	15 (50%)	
Education level			1.0
Bachelor’s degree	15 (50%)	15 (50%)	
Associate’s degree	15 (50%)	15 (50%)	
Initial assessment at enrollment	66.63 ± ±9.004	66.63 ± 9.042	0.275
Preliminary assessment of basic knowledge (40%)	32.57 ± 4.049	32.37 ± 4.760	0.861
Preliminary assessment of practical skills (40%)	24.13 ± 7.143	26.70 ± 7.212	0.171
Preliminary assessment of report interpretation (20%)	9.93 ± 2.815	10.13 ± 3.256	0.800

### Teaching strategy

#### Control group: traditional practical teaching model

The control group received traditional practical teaching. Instructors adhered strictly to the established curriculum, providing structured guidance. The model primarily combined theoretical instruction with laboratory demonstrations. Theoretical knowledge encompassing hematology testing principles, methods, and precautions was first delivered in the classroom, followed by standardized experimental demonstrations. Students then replicated procedures based on demonstrated steps under instructor supervision, with immediate correction of technical errors. Assessment focused primarily on the accuracy of experimental operations and theoretical knowledge grasp, with limited emphasis on clinical reasoning and laboratory result interpretation.

#### Experimental group: laboratory result-oriented modular teaching model

**Figure 1:**
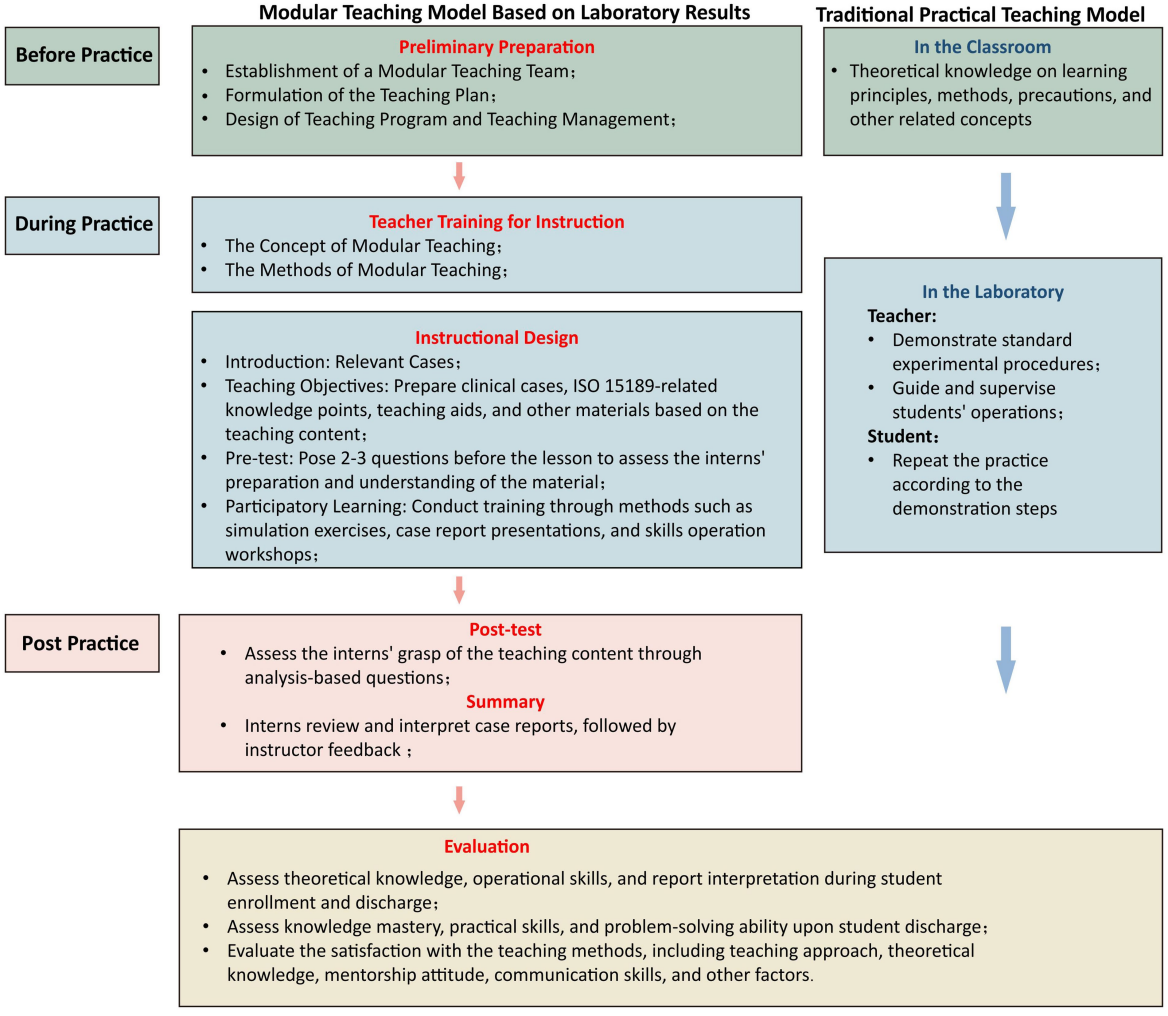
Schematic diagram of different teaching models for the experimental and control groups.

The experimental group’s curriculum integrated ISO 15189 principles. Training content was designed according to ISO 15189 requirements, departmental procedural documents, and the quality manual, incorporating key accreditation elements throughout. Implementation involved the following steps: (1) Preliminary preparation: A modular teaching team was established, comprising one team leader, one teaching secretary, and four instructors with >5 years of laboratory experience. The teaching secretary drafted the teaching plan, instructors implemented teaching activities and supervised plan execution, and the team leader oversaw plan design and process management. (2) Instructor training: Prior to teaching commencement, team members received training covering modular teaching concepts, instructional methods, and relevant content. (3) Teaching design: (i) Introduction: Three days before each weekly session, team members created training materials and introduced related cases aligned with teaching objectives to prepare students. (ii) Teaching objectives: Instructors prepared clinical cases, ISO 15189-related knowledge points, and teaching aids, clarifying expected knowledge and skill mastery. (iii) Pre-test: Instructors designed 2–3 content-related questions distributed with training materials 2 days in advance to assess preparation, identify weaknesses, and adjust teaching. (iv) Participatory learning: Methods such as simulations, case presentations, and skills workshops encouraged active participation. For instance, instrument training included ISO 15189-compliant calibration, maintenance, operation steps, and precautions, followed by supervised intern practice. Clinical case analysis followed ISO 15189 quality management processes to develop diagnostic reasoning skills. Interactive training enhanced conceptual understanding and engagement. (4) Post-test: One analytical question was provided post-session for interns to analyze and answer within a set timeframe, assessing comprehension and reinforcing learning. (5) Summary: Interns presented case reports using PowerPoint, reviewed and interpreted according to ISO 15189 standards. Instructor feedback addressed issues, further developing case analysis, clinical reasoning, and presentation skills (Figure 1).

### Evaluation criteria

**Figure 2:**
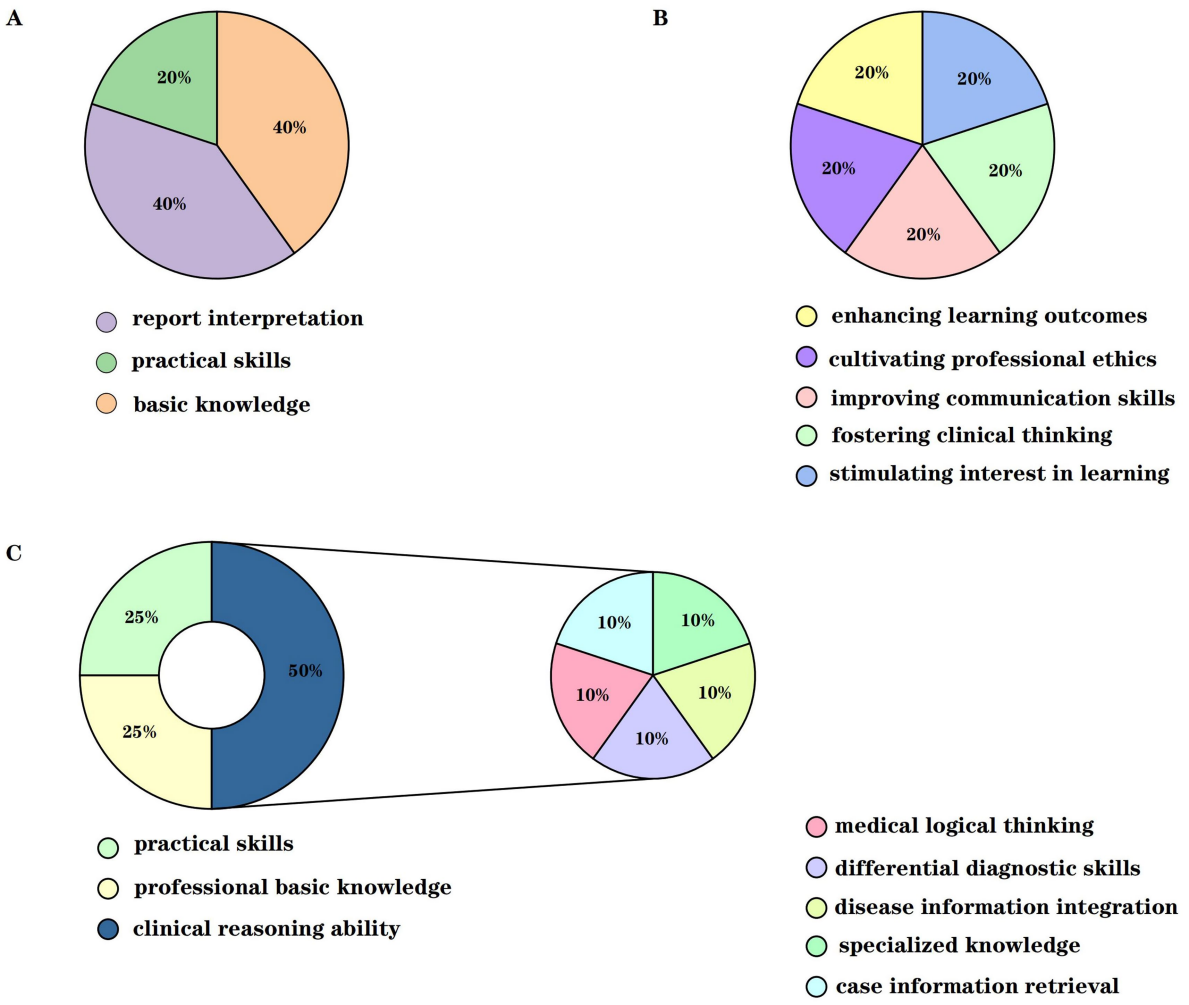
Schematic diagram of evaluation indicators for the experimental and control groups. **(A)** Represents the comprehensive evaluation scores prior to enrollment, which include the preliminary assessments of basic knowledge, practical skills, and report interpretation. **(B)** Represents the post-teaching evaluation of teaching effectiveness, which consists of five components: stimulating interest in learning, fostering clinical thinking, improving communication skills, cultivating professional ethics, and enhancing learning outcomes. **(C)** Represents the post-teaching assessment indicators for the experimental and control groups in three areas: professional basic knowledge, practical skills, and clinical reasoning ability.

Assessment was employed to evaluate teaching quality, guiding intern learning abilities, strategies, and overall instruction quality. Aligned with clinical hematology characteristics, assessment criteria were developed based on prior research: (i) Intern performance Evaluation: Upon entry, instructors assessed baseline performance and learning outcomes (100-point scale) covering knowledge acquisition, practical skills, and problem-solving abilities, enabling inter-group comparisons. (ii) Examination comparison: Performance in professional knowledge (100 points), operational skills (100 points), and clinical reasoning (100 points) was compared between groups. The clinical reasoning assessment evaluated case information retrieval, specialized knowledge, disease information integration, diagnostic/differential diagnostic skills, and medical logical thinking. (iii) Satisfaction survey: Both groups completed anonymous surveys rating satisfaction (100-point scale per item) with teaching methods, theoretical depth, instructor attitude, and communication skills. The overall satisfaction rate was calculated as a percentage (Figure 2).

### Statistical methods and data processing

Statistical analyses were performed using SPSS version 25.0. Normally distributed data are presented as mean ± standard deviation. Inter-group comparisons utilized independent-sample *t*-tests. A significance level of *p* < 0.05 was applied.

## Results

### Demographic information

All 60 interns (30 experimental group, 30 control group) completed the study. Demographic characteristics and pre-enrollment comprehensive evaluation scores showed no significant differences between groups (*p* > 0.05), confirming comparability (Table 1).

### Comparison of test results between the two groups of clinical laboratory interns after teaching

This study compared and analyzed the assessment results of clinical laboratory interns in the experimental and control groups across three dimensions: professional foundational knowledge, practical skills, and clinical reasoning ability. The results revealed that the teaching effectiveness was significantly superior in the experimental group compared to the control group. The evaluation scores across all dimensions were markedly higher in the experimental group, and these differences were statistically significant (*p* < 0.05) (see Table 2).

**Table 2:** Comparison of comprehensive skills scores after teaching.

Item	Control group (*n* = 30)	Experimental group (*n* = 30)	*p*
Professional basic knowledge test	87.00 ± 4.624	90.17 ± 4.356	0.008
Practical skills test	88.20 ± 4.604	92.87 ± 4.075	<0.001
Clinical thinking ability test	64.43 ± 5.008	86.47 ± 4.191	<0.001
Case information retrieval ability	13.43 ± 2.161	19.03 ± 0.999	<0.001
Specialized knowledge mastery ability	13.17 ± 2.086	17.13 ± 2.047	<0.001
Disease information integration ability	12.47 ± 1.756	16.23 ± 1.960	<0.001
Diagnostic and differential diagnosis ability	12.70 ± 1.915	16.90 ± 1.900	<0.001
Medical logical thinking ability	12.67 ± 1.845	17.17 ± 1.967	<0.001

Based on the data in Table 2, a detailed comparative analysis of the clinical reasoning ability indicators was conducted. The results demonstrated that the experimental group scored significantly higher on all clinical reasoning ability evaluation criteria than the control group (*p* < 0.05), indicating comprehensive enhancement in the core elements of clinical reasoning. Specifically, the experimental group exhibited notably stronger case information integration skills, suggesting that these interns could rapidly identify key information and conduct more effective analyses when confronted with clinical problems. Furthermore, the experimental group demonstrated an advantage in medical logical thinking, enabling them to make sound judgments based on clinical data and knowledge. The experimental group also outperformed the control group in key indicators such as diagnostic and differential diagnostic abilities, as well as mastery of professional knowledge. Overall, the significantly higher clinical reasoning ability scores of the experimental group confirmed that the laboratory result-oriented modular teaching model had a substantial positive impact on the clinical reasoning abilities of medical laboratory students.

### Comparison of teaching satisfaction between the two groups of interns

To comprehensively evaluate teaching satisfaction, the performance of the two intern groups was compared across five key dimensions: stimulating learning interest, fostering clinical thinking, improving communication skills, cultivating professional ethics, and enhancing learning outcomes (see Table 3). The results indicated that the experimental group expressed significantly higher satisfaction than the control group across all five dimensions (*p* < 0.05).

**Table 3:** Evaluation of the effect of teaching after the teaching session.

Item	Control group (*n* = 30)	Experimental group (*n* = 30)	*p*
Stimulate interest in learning	13.10 ± 2.354	16.47 ± 2.315	<0.001
Fostering clinical thinking	11.87 ± 1.592	17.27 ± 1.837	<0.001
Improving communication skills	13.03 ± 1.752	17.43 ± 1.794	<0.001
Cultivating professional ethics	14.23 ± 1.977	16.43 ± 2.542	<0.001
Enhancing learning outcomes	13.33 ± 2.264	16.60 ± 2.527	<0.001
Total score	65.57 ± 4.500	84.20 ± 5.242	<0.001

Regarding stimulating learning interest, interns in the experimental group generally found the teaching model novel and engaging, which significantly increased their learning motivation and intellectual curiosity. In terms of fostering clinical thinking, the experimental group reported a marked improvement in their clinical reasoning ability, which they attributed to the teaching approach integrating case analysis with practical exercises. Moreover, interns in the experimental group demonstrated greater initiative and efficiency when communicating with patients, instructors, and peers, indicating an improvement in their communication skills. In cultivating professional ethics, the experimental group exhibited a deeper understanding and better application of medical ethics principles. Finally, concerning the enhancement of learning outcomes, the experimental group surpassed the control group in both academic performance and clinical skills, reflecting the significant efficacy of the experimental teaching model.

## Discussion

Experimental medicine demands strong practical applicability and high professional standards, necessitating training approaches that effectively integrate theoretical knowledge with clinical practice. Contemporary laboratory education requires multidisciplinary professionals capable of interpreting complex diagnostic data, participating in clinical decision-making, and providing evidence-based recommendations (22–24). To address this need, we revised the internship syllabus by embedding ISO 15189 quality management principles throughout the curriculum. Routine work in the laboratory is closely linked to laboratory equipment and quality control; however, traditional syllabi often emphasize scheduling and foundational knowledge while underemphasizing critical operational competencies such as equipment troubleshooting, quality control implementation, and diagnostic interpretation (25). Therefore, these aspects were also incorporated into the training plan for interns, aiming to better prepare interns to become qualified and modern laboratory management professionals.

During implementation, theoretical and practical training were delivered concurrently through a laboratory result-oriented modular framework. This approach aligns with innovative pedagogical models documented in recent literature, such as virtual-physical hybrid courses in microbiology education (26) and outcome-based teaching in clinical laboratories (27). Our study demonstrated that interns trained under this ISO 15189-integrated modular model scored significantly higher in hematology theory assessments (*p* < 0.05) than those in traditional training. This improvement reflects the model’s capacity to contextualize theoretical knowledge through authentic laboratory outcomes—enhancing comprehension and retention. The experimental group also demonstrated superior practical competencies, including: (1) increased technical proficiency in procedures such as coagulation testing with improved error recognition; (2) enhanced analytical efficiency; and (3) strengthened diagnostic interpretation through multidimensional integration of hematologic parameters with clinical presentation.

In terms of practical skills, the experimental group interns performed better in aspects such as the accuracy of instrument operation, the accuracy and efficiency of test results, and overall performance. For example, in the operation of coagulation function testing, the experimental group interns were able to operate the instruments more proficiently and provide more reasonable explanations and preliminary judgments for abnormal test results. In terms of result analysis, the experimental group interns demonstrated stronger capabilities in the comprehensive analysis of blood test results. They were able to propose more reasonable diagnostic suggestions by integrating results from multiple blood tests with the patient’s clinical symptoms.

This improvement is attributed to the systematic study of various blood testing modules and result-oriented thinking training within the modular teaching model. Specifically, throughout the overall teaching process, the instructors employed a combination of observation and explanation to integrate ISO 15189’s competency requirements for laboratory personnel into the curriculum. Three key strategies significantly enhanced the students’ learning outcomes: First, students were encouraged to explore clinical knowledge and diseases autonomously, selecting the most appropriate methods to obtain clinical data and learn about disease diagnosis and treatment. This self-directed learning process not only improved the students’ clinical reasoning abilities but also helped them master the medical laboratory knowledge system more comprehensively. Second, the teaching content and practical training were divided into specific modules, and repeated practice in short time intervals reinforced the students’ learning, enabling them to internalize detailed operational knowledge and understand the theoretical basis behind the procedures, thus making their learning more systematic. Each module was supported by specialized instructors to ensure consistency and quality in the content, reducing the students’ learning pressure and enhancing their ability to learn independently. Lastly, this teaching model centered on laboratory test results. Under the guidance of instructors, interns were able to combine theoretical knowledge with real clinical cases. This integrated approach established an iterative learning process where theoretical knowledge informed practical application, subsequently deepening conceptual understanding - particularly regarding quality management systems essential for diagnostic reliability.

However, this study also has some limitations. It was conducted at a single tertiary hospital in Nanjing, and the sample size was relatively small, which may introduce regional biases. Future studies could expand the sample size and validate the model across different regions and hospitals of varying levels to further refine this teaching model. During the teaching process, although the model was based on laboratory results, some rare cases might not have been sufficiently covered in the curriculum. Future iterations of the teaching model could incorporate more rare case studies to broaden the interns’ perspectives. A paramount limitation is that the absence of longitudinal data prevents assessment of competency retention in professional practice, while the model shows significant promise in developing foundational competencies within the training period, longitudinal studies are needed to evaluate its true impact on career development and the quality of laboratory professionals produced (28). Future refinements should incorporate digital learning tools for rare case exposure and establish longitudinal tracking systems to correlate training experiences with workplace performance metrics.

## Conclusion

This study evaluated the implementation of a laboratory-result-oriented modular teaching model integrated with ISO 15189 principles for clinical hematology interns. Compared to traditional methods, the model demonstrated significantly enhanced teaching effectiveness, evidenced by statistically higher scores in theoretical examinations, operational skill assessments, and clinical reasoning evaluations (*p* < 0.05). Furthermore, interns reported significantly greater satisfaction with the teaching process. By utilizing actual laboratory test results as the learning anchor and embedding ISO 15189 requirements (covering personnel competence, procedural documentation, quality control, equipment management, and continuous improvement) within discrete modules, the model effectively bridges theoretical knowledge with the practical demands of standardized laboratory practice. Limitations include the single-center design and sample size, which necessitate further validation across diverse settings. Future research should also incorporate longitudinal studies to assess competency retention and explore the integration of digital tools for broader case exposure.

## Data Availability

The original contributions presented in the study are included in the article/Supplementary material, further inquiries can be directed to the corresponding authors.
