# Supplementary material for: Application of laboratory-result-oriented modular teaching model in the training of clinical hematology laboratory interns
Source: Front Med (Lausanne). 2025 Aug 18;12:1612934. doi: 10.3389/fmed.2025.1612934 (PMC12399513; doi:10.3389/fmed.2025.1612934)
Supplement: Supplementary file 1 [file Table_1.doc]

**Supporting Information**

**Application of Laboratory-Result-Oriented Modular Teaching Model in the Training of Clinical Hematology Laboratory Interns**

Hui Xie^a*^, Yijia Zhu^a*^, Lin Chen^a^, Aofan Wang^b^, Linyan Gong^c^, Tingting Tan^a^, Xian Wang^a†^, Yuxin Chen^a†^

^a^Department of Laboratory Medicine, Nanjing Drum Tower Hospital Clinical College of Nanjing Medical University, Nanjing, Jiangsu, China

^b^Department of Laboratory Medicine, Nanjing Drum Tower Hospital Clinical College of Jiangsu University, Zhenjiang, Jiangsu, China

^c^Department of Laboratory Medicine, Nanjing Drum Tower Hospital Clinical College of Nanjing University of Chinese Medicine, Nanjing, Jiangsu, China.

Hui Xie and Yijia Zhu contributed equally to this work. Author order was determined in order of the contribution to this study.

**Corresponding author**

Yuxin Chen, Department of Laboratory Medicine, Nanjing Drum Tower Hospital Clinical College of Nanjing Medical University, Nanjing, Jiangsu, China, Email:yuxin.chen@nju.edu.cn;

Xian Wang, Department of Laboratory Medicine, Nanjing Drum Tower Hospital Clinical College of Nanjing Medical University, Nanjing, Jiangsu, China, Email: ctnicat@163.com.

### ****Table 1: Clinical Reasoning Assessment Rubrics**** Based on ISO 15189 Competency Requirements

| **Item** | **Criteria for Judgement** | **Max Score** |
| --- | --- | --- |
| Case Information Retrieval Ability | Ability to integrate patients' medical history, clinical manifestations and multi-laboratory results. | 20 |
| Specialized Knowledge Mastery Ability | Ability to correctly apply the principles and methodological limitations of hematology detection. | 20 |
| Disease Information Integration Ability | Ability to make preliminary diagnosis based on laboratory data. | 20 |
| Diagnostic and Differential Diagnosis Ability | Key identification points for identifying similar diseases. | 20 |
| Medical Logical Thinking Ability | Integrity and causality of reasoning chain. | 20 |

### ****Table 2 Scoring Rubric for Dimension 1 - Case Information Retrieval Ability****

| **Level** | **Descriptors** | **Score** |
| --- | --- | --- |
| ****Excellent**** | Rapidly integrates **all critical information** (e.g., bleeding history, medication use) with multi-source laboratory data (CBC/coagulation/bone marrow) without omissions | 16-20 |
| ****Good**** | Identifies **core information** (e.g., anemia and thrombocytopenia) but misses one minor data point (e.g., medication history) | 11-15 |
| ****Adequate**** | **Fragmented information extraction** (isolated focus on single parameters like Hemoglobin(Hb), fails to establish clinical correlations | 6-10 |
| ****Inadequate**** | Omits **critical clinical information** (e.g., ignores cancer history), leading to misguided analysis | 0-5 |

### ****Table 3 Scoring Rubric for Dimension 2 - Specialized Knowledge Mastery Ability****

| **Level** | **Descriptors** | **Score** |
| --- | --- | --- |
| ****Excellent**** | Accurately explains **methodological interferences** (e.g., cold agglutinin effect on mean corpuscular volume(MCV) and proposes **ISO-compliant solutions** (e.g., 37°C incubation) | **16-20** |
| ****Good**** | Recognizes methodological limitations but provides **incomplete solutions** (e.g., identifies EDTA-dependent platelet clumping without recommending smear review) | **11-15** |
| ****Adequate**** | Only states **basic theories** (e.g., PT prolongation indicates extrinsic pathway disorder) without contextualizing to actual cases | **6-10** |
| ****Inadequate**** | **Misapplies knowledge** (e.g., misdiagnoses heparin contamination as DIC) | **0-5** |

### ****Table 4 Scoring Rubric for Dimension 3 - Disease Information Integration Ability****

| **Level** | **Descriptors** | **Score** |
| --- | --- | --- |
| ****Excellent**** | Proposes **≥2 evidence-based diagnoses** with: • **Direct diagnostic evidence** (e.g., reduced hemoglobin, increased mean corpuscular volume, and increased red blood cell distribution width all indicate megaloblastic anemia) • **ISO 15189-compliant annotations** (e.g., recommend serum folate or B12 testing) | **16-20** |
| ****Good**** | Proposes **1 valid diagnosis** but: • **Partial evidence gaps** (e.g.,diagnose anemia solely based on the decrease in hemoglobin levels, without considering the reticulocyte count, in order to rule out the possibility of hemolysis) • Omits **testing limitations** | **11-15** |
| ****Adequate**** | Provides **vague diagnostic direction** (e.g., anemia under investigation) or: • **Misinterprets key indicators** (e.g., attributes prothrombin time prolongation to liver disease instead of Factor VII deficiency) | **6-10** |
| ****Inadequate**** | **Misdiagnoses core data** (e.g., diagnoses EDTA-dependent pseudothrombocytopenia as idiopathic thrombocytopenic purpura) | **0-5** |

### ****Table 5 Scoring Rubric for Dimension 4 - Diagnostic and Differential Diagnosis Ability****

| **Level** | **Descriptors** | **Score** |
| --- | --- | --- |
| ****Excellent**** | Lists **≥3 differential diagnoses** with: • **Clear distinguishing criteria**  • **Prioritized testing strategy** | **16-20** |
| ****Good**** | Lists **2 differentials** but: •**Incomplete differentiation** (e.g., in disseminated intravascular coagulation, the level of D-dimer increases, but no mention of idiopathic thrombocytopenic purpura was made) • Lacks **risk-based prioritization** | **11-15** |
| ****Adequate**** | Merely **lists disease names** without comparative analysis (e.g., anemia can be classified into iron deficiency anemia, megaloblastic anemia, and aplastic anemia) | **6-10** |
| ****Inadequate**** | Proposes **contradictory differentials** (e.g., suggests anemia subtypes when Hb is normal) | **0-5** |

### ****Table 6 Scoring Rubric for Dimension 5 - Medical Logical Thinking Ability****

| **Level** | **Descriptors** | **Score** |
| --- | --- | --- |
| ****Excellent**** | Demonstrates **complete causal reasoning** with: • **Quality control integration** (e.g., first exclude hemolysis interference before analyzing hyperkalemia) • **Risk escalation protocol** (e.g., platelet count<10×10⁹/L requires immediate verbal report) | **16-20** |
| ****Good**** | Shows **logically sound reasoning** but: • Neglects **pre-analytical factors** (e.g., ignores effects of difficult venipuncture on coagulation) • Omits **ISO procedure references** (e.g., fails to mention reviewing instrument flags) | **11-15** |
| ****Adequate**** | Presents **disconnected conclusions** (e.g., directly diagnoses Disseminated Intravascular Coagulation without analyzing Fibrinogen or D-dimer) | **6-10** |
| ****Inadequate**** | Exhibits **faulty causality** (e.g., fever causes leukocytosis) | **0-5** |
